# Sliding tube-assisted ERCP in a patient who underwent double tract reconstruction anatomy after proximal gastrectomy

**DOI:** 10.1055/a-2145-1671

**Published:** 2023-08-21

**Authors:** Koichiro Kawano, Mamoru Takenaka, Reiko Kawano, Takao Katoh, Katsuhisa Nishi, Chang-Il Kwon, Masatoshi Kudo

**Affiliations:** 1 Department of Gastroenterology Hyogo Prefectural Awaji Medical Center, Sumoto, Hyogo, Japan; 2 Departments of Gastroenterology and Hepatology, Kindai University Faculty of Medicine, Osaka-Sayama, Osaka, Japan; 3 Digestive Disease Center, CHA Bundang Medical Center, CHA University School of Medicine, Seongnam, Republic of Korea

For patients with surgically altered gastrointestinal anatomy, the difficulty of performing endoscopic retrograde cholangiopancreatography (ERCP) has been reported 1 2 3 . After proximal gastrectomy, double tract reconstruction is usually performed (PG-DT), and some patients with PG-DT require ERCP. Although a technique of guidewire-assisted side-viewing scope insertion for patients with PG-DT has been reported 4 , the bent and tortuous nature of the interstitial jejunum and the end-to-side anastomosis of the gastric jejunum make it difficult ( Fig. 1 ).

**Fig. 1:**
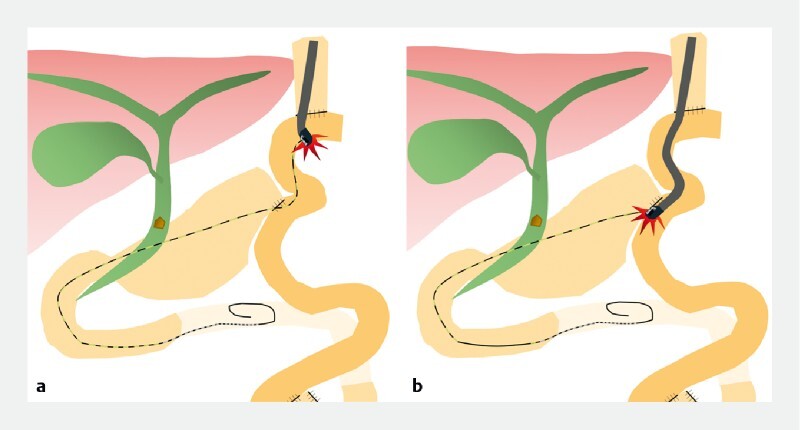
Although a technique of guidewire-assisted side-viewing scope insertion has been reported for endoscopic retrograde cholangiopancreatography in patients with proximal gastrectomy followed by double tract reconstruction, the bent and tortuous nature of the interstitial jejunum and the end-to-side anastomosis of the gastric jejunum make insertion of the side-viewing scope difficult.

A large-diameter sliding tube (ST-CB1; Olympus, Tokyo, Japan), which was designed for colonoscopy, has a length of 770 mm, and outer and inner diameters of 16.2 mm and 13.8 mm, respectively ( Fig. 2 ) 5 .

**Fig. 2:**
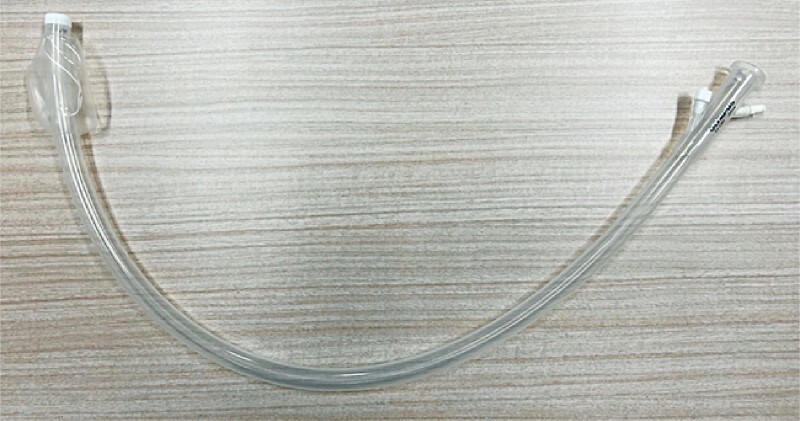
A large-diameter sliding tube (ST-CB1; Olympus, Tokyo, Japan), which was designed for colonoscopy, has a length of 770 mm, and outer and inner diameters of 16.2 mm and 13.8 mm, respectively.

Herein, we report a case of sliding tube-assisted ERCP using this single-use sliding tube in a patient with PG-DT.

A 63-year-old man who underwent PG-DT was admitted for treatment of cholelithiasis, for which ERCP was performed. First, guidewire-assisted side-viewing scope insertion was attempted, but the scope was unable to cross the gastrojejunal anastomosis owing to the flexion and meandering of the anastomosis. Therefore, we used a large-diameter sliding tube to secure the side-viewing scope insertion route. An upper endoscope was inserted into the gastric antrum beyond the gastrojejunal anastomosis, with a sliding tube attached to the scope; the scope was then removed, leaving the sliding tube in place ( Fig. 3 ).

**Fig. 3:**
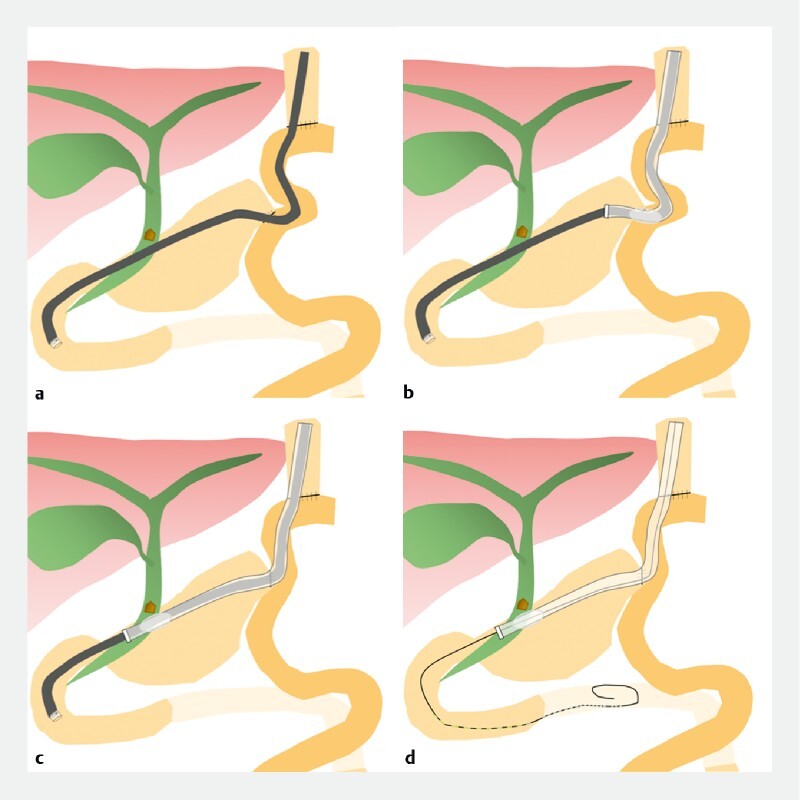
An upper endoscope was inserted into the gastric antrum beyond the gastrojejunal anastomosis, with a sliding tube attached to the scope; the scope was then removed, leaving the sliding tube in place.

The sliding tube straightened the curvature of the gastrojejunostomy lumen, and the side-viewing scope was successfully passed through the lumen of the tube to the duodenum; the scope stretch was also successful ( Fig. 4 ). Subsequently, removal of the stone in the common bile duct was successful ( Fig. 5 , Video 1 ). Adverse events, such as damage to the anastomotic site during insertion of the sliding tube, did not occur in this case.

**Fig. 4:**
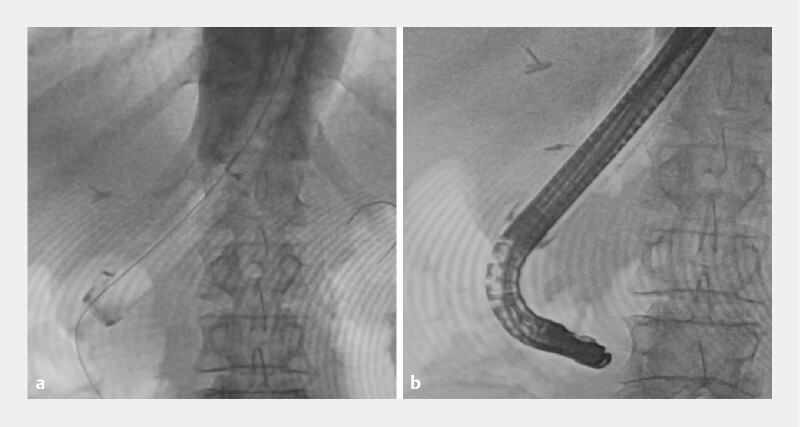
The sliding tube straightened the curvature of the gastrojejunostomy lumen, and the side-viewing scope was successfully passed through the lumen of the tube to the duodenum; the scope stretch was also successful.

**Fig. 5:**
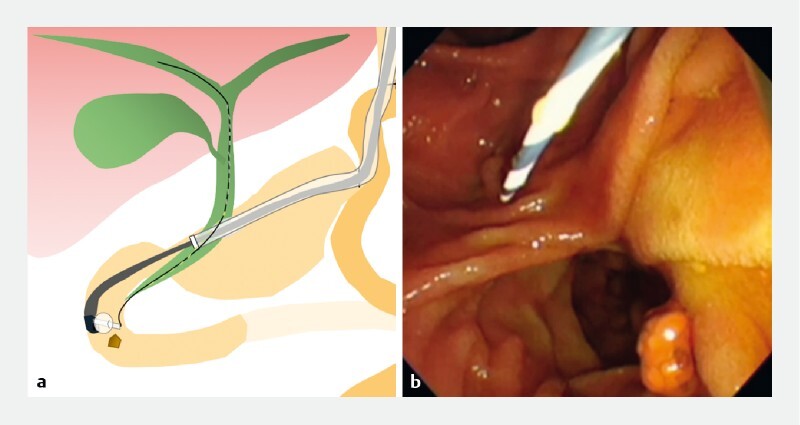
Removal of the common bile duct stone was successful.

**Video 1** Single-use sliding tube developed to improve colonoscopy operability for side-viewing scope insertion in a patient who had undergone proximal gastrectomy followed by double tract reconstruction.


This sliding tube-assisted side-viewing scope insertion technique is considered effective for patients with PG-DT anatomy.

Endoscopy_UCTN_Code_TTT_1AR_2AG

## Notes

Endoscopy E-Videos

https://eref.thieme.de/e-videos
*E-Videos* is an open access online section of the journal *Endoscopy* , reporting on interesting cases and new techniques in gastroenterological endoscopy. All papers include a high-quality video and are published with a Creative Commons CC-BY license. Endoscopy E-Videos qualify for HINARI discounts and waivers and eligibility is automatically checked during the submission process. We grant 100% waivers to articles whose corresponding authors are based in Group A countries and 50% waivers to those who are based in Group B countries as classified by Research4Life (see: https://www.research4life.org/access/eligibility/ ). This section has its own submission website at https://mc.manuscriptcentral.com/e-videos
